# Human Antibodies Can Cross Guinea Pig Placenta and Bind Its Neonatal Fc Receptor: Implications for Studying Immune Prophylaxis and Therapy during Pregnancy

**DOI:** 10.1155/2012/538701

**Published:** 2012-09-09

**Authors:** Evi Budo Struble, Li Ma, Lilin Zhong, A. Lesher, Joel Beren, Pei Zhang

**Affiliations:** ^1^Laboratory of Plasma Derivatives, Division of Hematology, OBRR, FDA, Bethesda, MD 20892, USA; ^2^Division of Veterinary Services, Center for Biologics Evaluation and Research, FDA, Bethesda, MD 20892, USA

## Abstract

Despite increased use of monoclonal and polyclonal antibody therapies, including during pregnancy, there is little data on appropriate animal models that could humanely be used to understand determinants of protection and to evaluate safety of these biologics in the mother and the developing fetus. Here, we demonstrate that pregnant guinea pigs can transport human IgG transplacentally at the end of pregnancy. We also observe that human IgG binds to an engineered soluble variant of the guinea pig neonatal Fc receptor *in vitro* in a manner similar to that demonstrated for the human variant, suggesting that this transplacental transport mirrors the receptor-based mechanism seen in humans. Using an intravenous antihepatitis B-specific immune globulin preparation as an example, we show that this transport results in neutralizing activity in the mother and the newborn that would potentially be prophylactic against hepatitis B viral infection. These preliminary data lay the groundwork for introducing pregnant guinea pigs as an appropriate model for the evaluation of antibody therapies and advancing the health of women and neonates.

## 1. Introduction

Vertical transmission of infectious agents during pregnancy has been documented and remains a problem for the fetus and the newborn. Depending on the time of infection, the sequelae of bacterial and viral transmission to the conceptus or fetus vary from fetal mortality/risk of deformities, to postnatal complications and failure to thrive. Given the immunological immaturity of the fetus, a robust immunological response to the invading pathogen in the pregnant woman would be an important determinant for improved outcome for mother and the newborn.

Although the status of the immune system during pregnancy remains incompletely understood, it is thought that alterations occur that include a dampening of innate responses and cell-mediated “Th1” adaptive immunity. In contrast, the humoral “Th2” adaptive immune responses seem to be enhanced [1]. It has been proposed that immune responses during pregnancy undergo “shifts” with the first and third trimesters being more “proinflammatory” and the second trimester showing characteristics that can be described as more “anti-inflammatory” [2]. These changes in immune responses are accompanied by changes in the rate and severity of bacterial and viral infections that occur during pregnancy. For example, the susceptibility to some, but not all bacterial, viral or other infections is increased during pregnancy [2, 3]. An increase in infectious disease severity and complications during pregnancy has been reported [4–6]. Increase of viremia is also seen in pregnant women chronically infected with hepatitis B [7, 8] and C [9]. As it becomes clear that we do not yet know how to improve antipathogen immunity in pregnant women, protecting the neonate from sequelae of infection by using safe prophylactic/therapeutic approaches remains a high priority.

Several specific (often referred to as hyperimmune) immune globulins have been approved by the FDA as safe and effective. These therapies are used in cases when active vaccination is not available, feasible, or effective, as well as a first line of treatment when risk of infection is high. There are reports of hyperimmune, for example, cytomegalovirus (CMV) [10], hepatitis B [11], and varicella [12] immune globulin preparations having been used off label during pregnancy to prevent congenital disease. A recent meta-analysis of publications in Chinese and English languages showed that administering HBIG products to pregnant women, in combination with maternal immunization, effectively reduces mother-to-child transmission of hepatitis B [13]. Active immunization of pregnant females has also resulted in better outcomes for their offspring. In several clinical and case control studies, immunization of pregnant women has been correlated with a decrease in hospitalization rates of their infants for influenza in the first 6 months of life [14] and prevented “approximately a third of all febrile respiratory illnesses in mothers and young infants” [15].

The mechanism underlying this reduction of adverse outcomes for the newborn is not completely known. It is generally assumed that improved prognosis results from a combination of the decrease in infectious agent load in the mother due to antibody administration or production and the acquiring of passive immunity in the fetus through transplacental transfer of the protective antibody.

At present, despite increased use of both mono- and poly-clonal antibody therapies, including during pregnancy, there is a paucity of data on appropriate animal models or species that could safely and humanely be used to understand determinants of protection and to evaluate safety of these preparations to the mother and the developing fetus. The most commonly used species, rats and rabbits, display differences in the rates of immunoglobulin transplacental transport that may limit their utility in such studies [16]. In this paper, we demonstrate that pregnant guinea pigs can effectively transport human IgG transplacentally at the end of pregnancy, thus providing an excellent model to evaluate the safety and efficacy of immune globulins.

## 2. Materials and Methods

### 2.1. Animal Study

All animal procedures were performed in a facility accredited by the Association for Assessment and Accreditation of Laboratory Animal Care International in accordance with protocols approved by the CBER Animal Care and Use Committee and the principles outlined in the 8th edition of the Guide for the Care and Use of Laboratory Animals by the Institute for Laboratory Animal Resources, National Research Council. Hartley Albino (Crl:HA) guinea pigs were purchased from Charles River Laboratories. The animals were housed in pairs or individually, and food and water were provided *ad libitum*. Female guinea pigs were mated in accordance with a published protocol [17] to produce timed pregnancies. Before test article administration, the animals were weighed and gently swaddled in a towel to provide comfort and immobilization. The lateral side of one rear leg was shaved, wiped with alcohol, and HBIGIV from a commercial source was administered intravenously on day 65 ± 2 of pregnancy via the saphenous vein at a dose of 100 IU/kg. Terminal blood samples were collected from anesthetized sows and piglets by cardiac puncture immediately following farrowing. Animals were then euthanized in a CO_2_ chamber while still under anesthesia.

### 2.2. Serum Processing and ELISA

Blood samples were stored overnight at 4°C to coagulate and then spun in a benchtop centrifuge at 1500 ×g for 5 minutes. Serum was collected, transferred into fresh tubes, and then frozen at −80°C for storage. Total and neutralizing antibody levels were determined by using Human IgG ELISA Kit (Bethyl Laboratories, Montgomery, TX, USA) and ETI-AB-AUK PLUS (DiaSorin, Saluggia, Italy), respectively. IgG subclasses were measured using human IgG subclass kits (Invitrogen). Each sample was run in duplicate and a standard curve was included in each plate.

### 2.3. Cloning of Soluble Guinea Pig FcRn

The sequences for guinea pig beta 2 microglobulin (B2M) and the alpha chain of the FcRn receptor (FCGRT) were synthesized *de novo *(OriGene USA, Rockville, MD, USA) from cDNA sequences obtained from Cavia porcellus genome in the *Ensembl server* database (http://useast.ensembl.org/Cavia_porcellus/Info/Index; transcript id *ENSCPOT00000002965* and *ENSCPOT00000026760*, resp.,). The codons in the DNA sequence for FCGRT were optimized for human cell expression by the supplier using proprietary technology and provided as inserts in pCMV entry vectors. These were transformed and amplified in *E. coli* using standard protocols, purified by appropriate kits (Qiagen Plasmid kits, Qiagen USA, Valencia, CA, USA) and sequenced to confirm that the DNA sequence encodes the correct primary sequence of the guinea pig FcRn protein. Primers were designed to allow for amplification of only the soluble domains but not the transmembrane domains of the guinea pig alpha and beta chains (Table 1). To identify these domains, guinea pig and human alpha and beta chains were aligned using BLAST and LALIGN algorithms from NCBI and ExPASy web servers (Figure 3). The plasmid for mammalian expression, pFUSE-Fc1 (InvivoGen USA, San Diego, CA, USA), was chosen to contain a strong secretion signal (Ile2) [18] which replaced the endogenous translocation signal of the FcRn alpha chain. For increased production and improved folding of the soluble protein, an approach that would allow secretion of a single chain soluble receptor containing the B2M and FCGRT segments separated by a linker of three GGGGS sequences, as described [19], was chosen. This approach, known as “overlapping PCR” or “splicing by overlap extension PCR,” involved two steps. First, the soluble domains of the alpha and beta chains were amplified separately using B2M and FCGRT forward and reverse primer sets, respectively (sequence of primers shown in Table 1). Following 30 cycles of PCR and double-stranded DNA purification (kits from New England Biolabs or equivalent), the two products were mixed in a second-step PCR reaction involving the two “outside” primers, that is, B2M forward and FCGRT reverse. As shown in Table 1, two variants of FCGRT reverse primer were designed. The variant without the stop codon would result in production of the soluble receptor conjugated in the C-terminus to Fc tag. The size of the construct was confirmed by agarose gel electrophoresis. After purification of the DNA and enzymatic digestion with EcoRI and BglII (New England Biolabs), the construct was inserted into the pFUSE plasmid for mammalian cell expression. Large amounts of this construct were prepared by standard methods, and the DNA amount was determined by UV absorption.

### 2.4. Constructing Stable Cell Lines

A suspension of Huh7 human liver cells was prepared in GlutaMAX DMEM medium supplemented with 10% FBS (Invitrogen Life Technologies USA, Grand Island, NY, USA) at a density of 2 × 10^5^ cells/mL, plated in a 6-well plate and incubated at 37°C in a CO_2_ incubator. The following day the growth medium was replaced by Opti-MEM serum-free medium mixed with Lipofectamine 2000 (Invitrogen) and 4 *μ*g pFUSE plasmid containing the single-chain soluble guinea pig FcRn DNA. The cells were placed in the 37°C CO_2_ incubator overnight and then passaged into a new plate after 1 : 3 dilution with DMEM/FBS. After surface adhesion (approximately 2–6 hours) zeocin (InvivoGen) was added to a final concentration of 100 *μ*g/mL. Resistant cells were obtained in about two weeks. To confirm secretion of the receptor, 2 mL of the cell culture medium for the chimeric (Fc-containing) construct was mixed with 50 uL slurry containing magnetic beads conjugated to protein-G (Invitrogen), allowed to bind for 15 minutes, washed with PBS containing 0.01% Tween 20 and then eluted with 30 *μ*L glycine HCl, pH 2.2. The entire amount was analyzed by western blot.

### 2.5. Production of Guinea Pig Soluble FcRn and Binding to Human IgG Column

To produce soluble guinea pig receptor, stably transfected Huh7 cells were grown in T-75 culture flasks (Fisher Scientific, Pittsburgh, PA, USA) in GlutaMAX DMEM supplemented with 10% FBS until they reached confluence, at which point the medium was replaced with BD Cell MAB serum free medium (Fisher) containing zeocin. The cultures were kept at 37°C in a CO_2_ incubator for two weeks at which point the growth medium was collected and its pH adjusted to 6.0 with 1 M HCl. The solution was loaded by gravity flow onto a column of 1 mL IgG Sepharose 6 Fast Flow resin (GE Healthcare Life Sciences, USA) that was packed, washed, and equilibrated with binding buffer containing 50 mM Na phosphate pH 6, 150 mM NaCl and 0.005% Tween 20. After washing with 3 mL binding buffer, the column was eluted with elution buffer containing 50 mM Tris Cl pH 8.5 and 150 mM NaCl. The load, flow-through, and eluate were analyzed by western blot.

### 2.6. Western Blots

Mixtures of sample and loading buffer, with or without reducing agent, were heated at 72°C for 7 minutes (if reducing agent was added) and loaded in NuPAGE MOPS 4–12% minigel (Invitrogen). The gel was run at constant voltage (120 V) for about 1 hour and then transferred onto a nitrocellulose membrane with an iBlot Dry Blotting System (Invitrogen), 10 minutes transfer time. After transfer, the membrane was blocked overnight at 4°C with shaking in blocking buffer containing 5% skim milk and 0.2% Tween in PBS. Rabbit polyclonal anti-FCGRT primary antibody (Proteintech USA) was diluted at an appropriate concentration in blocking buffer and allowed to bind to the membrane for 30 minutes at room temperature with shaking. After extensive washing with 0.2% Tween PBS solution (PBST), the membrane was transferred into a solution containing HRP-conjugated goat anti-rabbit secondary antibody (KPL, Gaithersburg, MD, USA) in blocking buffer and allowed to bind for 30 minutes at room temperature. After extensive washing with PBST, substrate mixture was prepared in accordance to manufacturer's instructions (Thermo Scientific West Pico Substrate, Fisher Scientific) and the membrane developed and imaged on an X-ray film or scanned with an imager (Kodak Image Station 4000 mm).

## 3. Results and Discussion

### 3.1. *In Vivo* Human Antibody Transplacental Transfer

**Figure 1:**
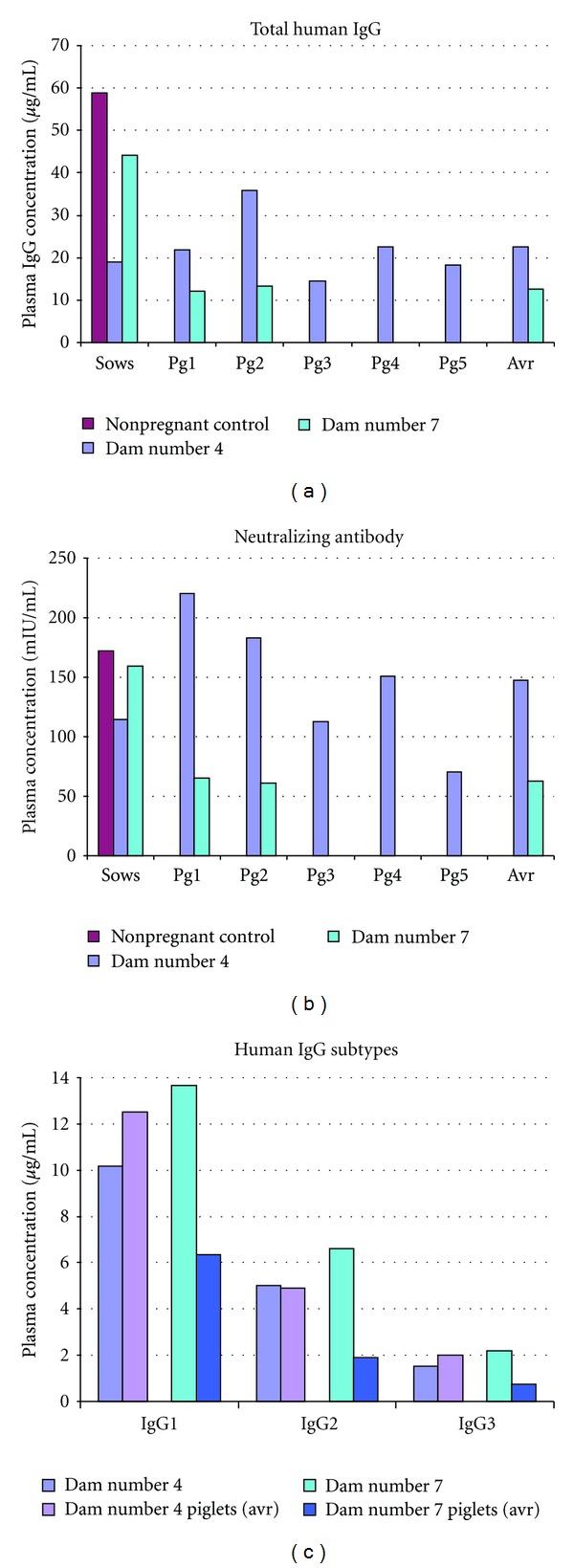
Human IgG transfer to the piglets of pregnant guinea pigs injected with a human immune globulin preparation. Two pregnant sows, numbered 4 and 7, were injected with HBIGIV on days 62 and 66 of pregnancy, respectively. Total and neutralizing human IgG, and the IgG subclasses ((a), (b), and (c), resp.) in the serum of the dams and the piglets (Pg1–5 and Pg1-2, resp.) soon after farrowing (on days 67 and 68, resp.) was quantified with commercial kits. All the piglets had human IgG in their blood stream. The concentrations for total (a) and neutralizing (b) antibody as well as for all the subtypes (c) were higher in the progeny of dam number 4, which delivered five days after the HBIGIV administration, than in that of dam number 7, which delivered two days following administration.

We detected circulating human antibodies in the serum of piglets of timed pregnant guinea pigs administered HBIGIV, demonstrating for the first time that the pregnant guinea pig could be utilized as a model to measure transplacental transfer of IgG in human immune globulin preparations. Dam number 4, which received 100 IU/kg HBIGIV on gestation day 62, delivered a litter of 5 piglets with circulating human antibodies on gestation day 67 (Figure 1). Another guinea pig, numbered 7 in our experiment, injected on day 66 of pregnancy with the same dose, delivered two piglets on day 68, both of which had human IgG in their serum. Both sows and their piglets had measurable amounts of IgG1, 2 and 3, but not of IgG4 (Figure 1(c)), likely due to the low concentration of IgG4 in the preparation used. The distribution of the IgG subclasses in the mothers, offspring, and the HBIGIV was very similar (data not shown).

These offspring of pregnant guinea pigs given HBIGIV near the term of pregnancy exhibited serum concentrations of human neutralizing antibody higher than 10 mIU/mL, the level that has been correlated with protection from HBV infection. Administering 100 IU/kg HBIGIV to pregnant sows results in titers >50 mIU/mL in the sow and the offspring, that is, more than five times the titer associated with protection from HBV (Figure 1(b)).

In our study, as expected, piglets born to the guinea pig exposed to human antibodies for five days (dam number 4) showed higher levels of human antibodies in their serum than did the progeny of guinea pig number 7, which was exposed to the antibody preparation for only two days. These piglets also had, on average, a higher total concentration of human antibodies in their serum than did their dams. This was the case for IgG1 and 3, but not for IgG2, indicating that IgG2 may be slightly less efficiently transferred than the other two subtypes, as is also seen in humans. Although more data are needed to strengthen the evidence, this observation suggests that, as in humans, the transfer of human antibodies in guinea pigs is a unidirectional, receptor-driven process that can result in fetal concentrations that are higher than maternal. Although guinea pigs are expected to make antibodies to human IgG, we did not detect any in these animals. Given that these guinea pigs were exposed to a single dose of human IgG for a short time (two and five days), this observation is in keeping with the kinetics of humoral immune response and with data from other species, such as Balb/c mice. In the later, immune-mediated clearance of the human IgG was only seen on day six [20].

The transfer of human antibodies transplacentally in pregnant guinea pigs is not unexpected. Compared to that of other small laboratory animals, the reproductive system of the female guinea pig most closely resembles that of humans, especially in the morphology and physiology of the placenta, the relative length of pregnancy and the development of the young at the time of delivery. Like humans, guinea pigs have a hemochorial placenta, where the chorion, or membrane enclosing the fetus, comes in direct contact with the mother's blood. Such a placenta displays a transport barrier similar to that of the human placenta [21]. Pregnant guinea pigs display key hormonal characteristics of parturition, develop spontaneous toxemia and preeclampsia during pregnancy, and have been proposed as the best small animal model of human pregnancy [22] and for performing developmental and reproductive toxicology (DART) studies to support regulatory applications [16, 23]. The guinea pig has been used for transplacental transfer studies of nutrients such as amino acids and glucose [24, 25]. It has even been shown that homologous and heterologous guinea pig antibodies [26, 27] as well as a commercial humanized IgG4 antibody [16] do transfer from pregnant sows to their fetuses. Most importantly, the transfer of antibodies from mother to the embryo occurs during the last third of pregnancy [28], in striking resemblance to humans, especially when compared to the different pattern observed in other rodents such as rats [29] and likely in mice, where the transfer is primarily via the GI tract and after delivery.

The process of IgG transplacental transfer in humans as a mechanism to confer protective immunity from the mother to the infant has been postulated and suspected for a long time [30–33]. Experimental observations on immunoglobulin half life [34, 35], gastric absorption in suckling rat [29], and intrauterine transport to the fetus [36], prompted Brambell to hypothesize that these processes are mediated by the same receptor [37]. The receptor responsible for this transfer was first cloned from murine gut epithelium [38] and its similarity to the MHC class I molecules noted. It was subsequently shown that this receptor, named FcRn (for Fc receptor neonatal), is expressed in human placental syncytiotrophoblasts [39], but direct evidence for its role in IgG transplacental transfer was not obtained until much later [40, 41].

Morphologically, FcRn receptor is a heterodimer composed of the alpha chain, known as Fc fragment of IgG, receptor, transporter, alpha (FCGRT, often interchangeably referred to as FcRn), and the beta chain. This latter chain, known as beta 2 microglobulin (B2M), is a protein that also forms heterodimers with MHC class I proteins. It has been shown that, in Madin-Darby canine kidney cells, both FCGRT and B2M molecules are required for functional expression of the FcRn [42]. When transfected alone, the alpha chain appears not to be correctly folded and does not function *in vitro*. However, both FcRn expression and function are recovered if B2M is cotransfected with the alpha chain.

Following the first crystal structure of the receptor in complex with Fc [43], elegant structural and molecular biology studies (for a review see [44]) have elucidated the molecular determinants of the physiologic function of the FcRn. Briefly, FcRn binds IgG at acidic pH (pH < 6.5) in the endosome, rescuing it from catabolic digest. This binding is negligible at neutral or higher pH, resulting in the release of IgG into the extracellular medium. When carried out by the receptor expressed in endothelial cells, this affects IgG release into the circulation and enhances antibody half life. In the case of the gut epithelium of suckling rodent pups, this results in IgG from maternal milk being transcytosed from the apical to the basolateral side of the GI tract [45]. A similar process is thought to occur across the human syncytiotrophoblast, allowing maternal antibodies to be transported across the placenta to the fetus. The molecular basis of this exquisite pH dependency has been identified to be a “histidine patch” in the C_H_2–C_H_3 regions of the IgG molecule that would be protonated at acidic pH but neutral under physiologic conditions [46]. At acidic pH, this patch on the IgG surface is involved in electrostatic interactions with glutamic and aspartic acid residues on FcRn and, together with specific hydrophobic contacts between the two molecules, provides the energy for binding and the observed nanomolar affinity.

**Figure 2:**
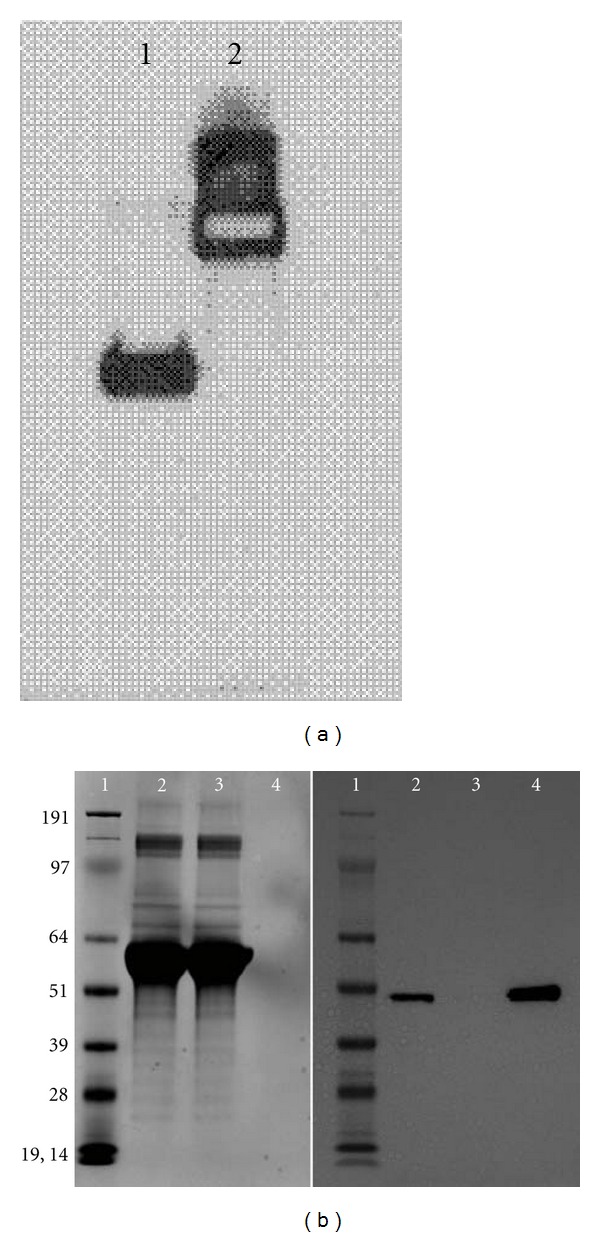
Expression and binding of engineered guinea pig soluble FcRn receptor. (a) This western blot confirms secretion of the guinea pig soluble FcRn by the Huh7 human liver cell line transiently transfected with pFUSE containing soluble FcRn. Shown are reduced (lane 1) and nonreduced (lane 2) guinea pig soluble receptor conjugated to Fc and blotted with rabbit anti-FCGRT polyclonal primary antibody specific for a FcRn alpha chain peptide partially conserved in the guinea pig sequence (see Figure 3(a) for the sequence). (b) Images of Coomassie stained gel (left) and a western blot demonstrating binding of the engineered guinea pig soluble FcRn receptor to a commercial human IgG column. Shown are the molecular weight markers (lane 1, with the respective mass shown), cell culture medium, column load (lane 2), column flow-through (lane 3), and column eluate (lane 4). The apparent molecular mass is lower than 50 kDa, whereas the theoretical molecular mass without glycosylation is ~43 kDa. The column binding and elution was repeated three times with similar results.

**Figure 3:**
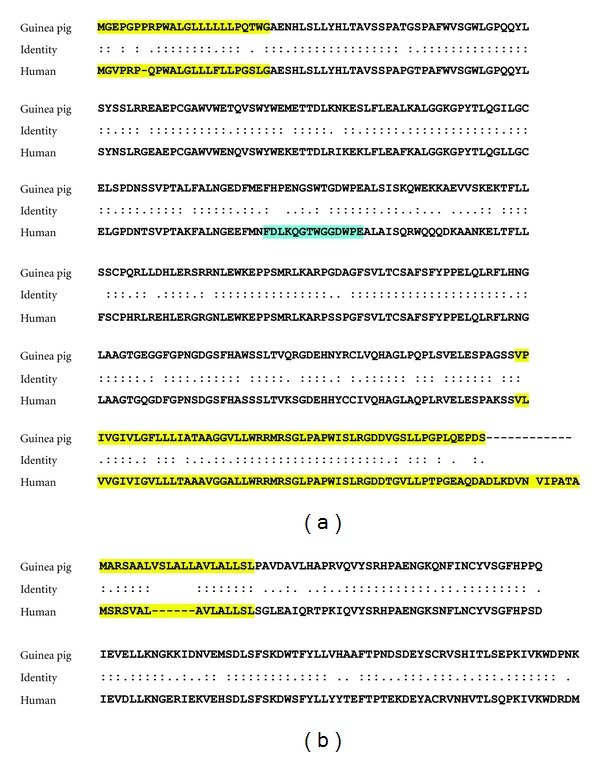
Alignment of human and guinea pig FCGRT (a) and B2M (b) chains of the FcRn receptor. Program LALIGN with matrix BLOSUM50, as described [48] was used. Similar results were obtained using BLAST. Regions highlighted in yellow correspond to signal and transmembrane domains of these chains and were not included in the engineered soluble receptor. The immunogen used for polyclonal anti-FCGRT antibody production is highlighted in cyan.

To demonstrate that guinea pig FcRn receptor specifically binds to human IgG, we decided to synthesize a soluble version of this receptor and perform *in vitro* binding, as it has been demonstrated for the human receptor [19, 47]. Using bioinformatics tools, we designed a soluble version of the guinea pig FcRn, synthesized it as a single chain by means of overlapping PCR, and expressed it in mammalian cells (Figure 2(a)). This version of the receptor was applied onto a human IgG column at acidic pH. The column completely absorbed the soluble guinea pig receptor from the medium, resulting in undetectable levels of the receptor in the column flow-through (Figure 2(b)). The bound receptor was released from the beads by applying elution buffer (pH 8–8.5), in a fashion analogous to that reported for a similar human FcRn construct [19]. This experiment demonstrated that the engineered guinea pig soluble FcRn protein we produced, and likely the membrane bound mature receptor, binds to human IgG, providing further evidence that the transplacental transfer of human antibodies we observed in pregnant guinea pigs is mediated by the FcRn receptor.

## 4. Conclusion

Our current studies demonstrate that transplacental transport of human IgG occurs in pregnant guinea pigs receiving an intravenous immune globulin preparation in the last third of pregnancy. We show that IgG subclasses one through three are measurable in both the sows and their piglets. Together with existing data indicating that IgG4 can be transferred transplacentally in the guinea pig [16], this finding provides preliminary evidence that all human IgG subclasses can pass guinea pig placenta at the last third of pregnancy.

Virus neutralizing activity was detectable in piglets at levels consistent with the target range for prophylaxis in human neonates.

In addition, *in vitro* binding data confirmed that engineered soluble guinea pig FcRn receptor binds human IgG similarly to the human soluble receptor.

## Figures and Tables

**Table 1:** Primers used to clone single-chain, soluble guinea pig FcRn. Underlined sequences correspond to EcoRI and BglII enzymatic sites in the B2M and FCGRT primers, respectively, whereas the sequences shown in bold face correspond to overlapping sequences in the overlapping PCR reaction which allow B2M and FCGRT to join in one segment separated by three GGGS linker sequences (in italics, see text for description).

Name	Primer DNA sequence	Primer corresponding peptide sequence
B2M forward	CTGGAATTCGCCGGCTGTGGACGCCGTCCTC	W N S P A V D A V L
B2M reverse	**GCCACCTCCGCC**TGAACCGCCTCCACCCTTGTTTGGATCCCATTT	K W D P N K *G G G G S * ***G G G G ***
FCGRT forward	**GGCGGAGGTGGC**TCTGGCGGTGGCGGATCAGCCGAGAACCACCTGAGCCTGCTGTACC	***G G G G*** * S G G G G S* A E N H L S L L Y
FCGRT reverse	CAGAGATCTGCTGCTGCCGGCGGGGCTCTCCAGCTCC	E L E S P A G S S R S L
FCGRT reverse with stop codon	CAGAGATCTCTATTAGCTGCTGCCGGCGGGGCT	S P A G S S **Stop** **Stop ** R S L

## References

[1] Whitacre CC, Reingold SC, O'Looney PA (1999). Biomedicine: a gender gap in autoimmunity. *Science*.

[2] Mor G, Cardenas I (2010). The immune system in pregnancy: a unique complexity. *American Journal of Reproductive Immunology*.

[3] Okoko BJ, Enwere G, Ota MOC (2003). The epidemiology and consequences of maternal malaria: a review of immunological basis. *Acta Tropica*.

[4] Styrt B, Sugarman B (1991). Estrogens and infection. *Reviews of Infectious Diseases*.

[5] Abrams ET, Miller EM (2011). The roles of the immune system in Women's reproduction: evolutionary constraints and life history trade-offs. *American Journal of Physical Anthropology*.

[6] Price ME, Fisher-Hoch SP, Craven RB, McCormick JB (1988). A prospective study of maternal and fetal outcome in acute Lassa fever infection during pregnancy. *British Medical Journal*.

[7] Hieber JP, Dalton D, Shorey J, Combes B (1977). Hepatitis and pregnancy. *Journal of Pediatrics*.

[8] Leung N (2009). Chronic hepatitis B in Asian women of childbearing age. *Hepatology International*.

[9] Honegger J, Prasad M, Colombo D, Bowen D, Walker C (2008). HCV-specific T-cell responses during acute Hepatitis C Virus infection in pregnancy. *Retrovirology*.

[10] Nigro G, Adler SP, La Torre R (2005). Passive immunization during pregnancy for congenital cytomegalovirus infection. *New England Journal of Medicine*.

[11] Kanai K, Takehiro A, Noto H (1985). Prevention of perinatal transmission of hepatitis B virus (HBV) to children of e antigen-positive HBV carrier mothers by hepatitis B immune globulin and HBV vaccine. *Journal of Infectious Diseases*.

[12] Chapman S, Duff P (1993). Varicella in pregnancy. *Seminars in Perinatology*.

[13] Shi Z, Li X, Ma L, Yang Y (2010). Hepatitis B immunoglobulin injection in pregnancy to interrupt hepatitis B virus mother-to-child transmission-a meta-analysis. *International Journal of Infectious Diseases*.

[14] Benowitz I, Esposito DB, Gracey KD, Shapiro ED, Vázquez M (2010). Influenza vaccine given to pregnant women reduces hospitalization due to influenza in their infants. *Clinical Infectious Diseases*.

[15] Zaman K, Roy E, Arifeen SE (2008). Effectiveness of maternal influenza immunization in mothers and infants. *New England Journal of Medicine*.

[16] Pentsuk N, van der Laan JW (2009). An interspecies comparison of placental antibody transfer: new insights into developmental toxicity testing of monoclonal antibodies. *Birth Defects Research. Part B*.

[17] Elvidge H (1972). Production of dated pregnant guinea pigs without postpartum mating. *Journal of the Institute of Animal Technicians*.

[18] Bamford RN, DeFilippis AP, Azimi N, Kurys G, Waldmann TA (1998). The 5’ untranslated region, signal peptide, and the coding sequence of the carboxyl terminus of IL-15 participate in its multifaceted translational control. *Journal of Immunology*.

[19] Feng Y, Gong R, Dimitrov DS (2011). Design, expression and characterization of a soluble single-chain functional human neonatal Fc receptor. *Protein Expression and Purification*.

[20] Zuckier LS, Georgescu L, Chang CJ, Scharff MD, Morrison SL (1994). The use of severe combined immunodeficiency mice to study the metabolism of human immunoglobulin G. *Cancer*.

[21] Kaufmann P, Davidoff M (1977). The guinea-pig placenta. *Advances in Anatomy, Embryology, and Cell Biology*.

[22] Mitchell BF, Taggart MJ (2009). Are animal models relevant to key aspects of human parturition?. *American Journal of Physiology. Regulatory Integrative and Comparative Physiology*.

[23] Rocca MS, Wehner NG (2009). The guinea pig as an animal model for developmental and reproductive toxicology studies. *Birth Defects Research. Part B*.

[24] Hill PMM, Young M (1973). Net placental transfer of free amino acids against varying concentrations. *Journal of Physiology*.

[25] Jansson T, Persson E (1990). Placental transfer of glucose and amino acids in intrauterine growth retardation: studies with substrate analogs in the awake guinea pig. *Pediatric Research*.

[26] Barnes JM (1959). Antitoxin transfer from mother to foetus in the guinea-pig. *The Journal of Pathology and Bacteriology*.

[27] Kulangara AC, Schechtman AM (1963). Do heterologous proteins pass from mother to fetus in cow, cat and guinea pig?. *Proceedings of the Society For Experimental Biology and Medicine*.

[28] Leissring J, Anderson J (1961). The transfer of serum proteins from mother to young in the guinea pig. I. Prenatal rates and routes. *The American Journal of Anatomy*.

[29] Halliday R (1955). Prenatal and postnatal transmission of passive immunity to young rats.. *Proceedings of the Royal Society of London. Series B*.

[30] Aycock WL, Kramer SD (1930). Immunity to poliomyelitis in mothers and the newborn as shown by the neutralization test. *The Journal of Experimental Medicine*.

[31] Du Pan RM, Wenger P, Koechli S, Scheidegger JJ, Roux J (1959). The passage of labelled *γ*-globulin through the human placenta. *Clinica Chimica Acta*.

[32] Gitlin D, Kumate J, Urrusti J, Morales C (1964). Selective and directional transfer of 7S *γ*2-globulin across the human placenta. *Nature*.

[33] Wiener A (1948). Rh factor in immunological reactions. *Annals of Allergy*.

[34] Fahey JL, Sell S (1965). The immunoglobulins of mice. V. the metabolic (catabolic) properties of five immunoglobulin classes. *The Journal of experimental medicine*.

[35] Waldmann TA, Strober W (1969). Metabolism of immunoglobulins. *Progress in Allergy*.

[36] Brambell FW, Hemmings WA, Henderson M, Rowlands WT (1950). The selective admission of antibodies to the foetus by the yolk-sac splanchnopleur in rabbits. *Proceedings of the Royal Society of London. Series B*.

[37] Brambell FW (1966). The transmission of immunity from mother to young and the catabolism of immunoglobulins. *Lancet*.

[38] Simister NE, Mostov KE (1989). An Fc receptor structurally related to MHC class I antigens. *Nature*.

[39] Simister NE, Story CM, Chen HL, Hunt JS (1996). An IgG-transporting Fc receptor expressed in the syncytiotrophoblast of human placenta. *European Journal of Immunology*.

[40] Firan M, Bawdon R, Radu C (2001). The MHC class I-related receptor, FcRn, plays an essential role in the maternofetal transfer of *γ*-globulin in humans. *International Immunology*.

[41] Ghetie V, Ward ES (2000). Multiple roles for the major histocompatibility complex class I-related receptor FcRn. *Annual Review of Immunology*.

[42] Claypool SM, Dickinson BL, Yoshida M, Lencer WI, Blumberg RS (2002). Functional reconstitution of human FcRn in Madin-Darby canine kidney cells requires co-expressed human *β*2-microglobulin. *Journal of Biological Chemistry*.

[43] Burmeister WP, Huber AH, Bjorkman PJ (1994). Crystal structure of the complex of rat neonatal Fc receptor with Fc. *Nature*.

[44] Roopenian DC, Akilesh S (2007). FcRn: the neonatal Fc receptor comes of age. *Nature Reviews Immunology*.

[45] Jones EA, Waldmann TA (1972). The mechanism of intestinal uptake and transcellular transport of IgG in the neonatal rat. *Journal of Clinical Investigation*.

[46] Raghavan M, Bonagura VR, Morrison SL, Bjorkman PJ (1995). Analysis of the pH dependence of the neonatal Fc receptor/immunoglobulin G interaction using antibody and receptor variants. *Biochemistry*.

[47] Chaudhury C, Brooks CL, Carter DC, Robinson JM, Anderson CL (2006). Albumin binding to FcRn: distinct from the FcRn-IgG interaction. *Biochemistry*.

[48] Myers EW, Miller W (1988). Optimal alignments in linear space. *Computer Applications in the Biosciences*.

